# A Pilot-scale Benthic Microbial Electrochemical System (BMES) for Enhanced Organic Removal in Sediment Restoration

**DOI:** 10.1038/srep39802

**Published:** 2017-01-06

**Authors:** Henan Li, Yan Tian, Youpeng Qu, Ye Qiu, Jia Liu, Yujie Feng

**Affiliations:** 1 State Key Laboratory of Urban Water Resource and Environment, Harbin Institute of Technology, No 73 Huanghe Road, Nangang District, Harbin 150090, China; 2 Heilongjiang Academy of Chemical Engineering, No 3, Nanhu Street, Century District, High-Tech Zone, Harbin 150028, Heilongjiang, China; 3 School of Life Science and Technology, Harbin Institute of Technology, No. 2 Yikuang Street, Nangang District, Harbin 150080, China

## Abstract

A benthic microbial electrochemical systems (BMES) of 195 L (120 cm long, 25 cm wide and 65 cm height) was constructed for sediment organic removal. Sediment from a natural river (Ashi River) was used as test sediments in the present research. Three-dimensional anode (Tri-DSA) with honeycomb structure composed of carbon cloth and supporting skeleton was employed in this research for the first time. The results demonstrated that BMES performed good in organic-matter degradation and energy generation from sediment and could be considered for river sediments *in situ* restoration as novel method. Community analysis from the soil and anode using 16S rDNA gene sequencing showed that more electrogenic functional bacteria was accumulated in anode area when circuit connected than control system.

Sediments are an important component of aquatic environment. As repositories of the overlying water body (e.g., oceans, lakes, rivers, or reservoirs), sediments are usually composed of organic and inorganic materials and shelter a complex microbial ecosystem that thrives on several different electron donors and acceptors^1^,^2^.

Given the fact of long-term drainage of industrial wastewater and municipal sewage without treatment or not meeting the set treatment standards, large amounts of pollutants and nutrients have been deposited onto the sediments, such as organic matter, nitrogen, and phosphorus and thereby potentially threatening the ecosystem integrity^3^. It is becoming a serious problem and an enormous task in many counties in the world to recover the river ecological function. For example, Danube river, which was once severely polluted, took the effort of GEF (Global Environment Facility) and EU countries more than 10 years to restore the watershed ecologically. During the 10 years, large quantities of investment was used from EU countries for this huge restoration project. In China, watershed pollution existed for long time because of the following two reasons, (1) the historic accumulated pollutants during the past 60 years with the industrial development and urbanization and (2) the fact that there are still some new pollutants input into the natural water body every year.

River recovery to its natural state usually needs a long time even when there is not new pollutants input into the system. So remediation techniques, which can accelerate the restoration rate are gaining importance globally. These methods include physical, chemical and biological processes. River dredging or using bio-agents is common for river and sediment remediation, yet low efficiencies and high costs are the two bottlenecks^4^. Most recent evidence indicates that the disposal costs of sludge account for 25–60% of the total cost of wastewater treatment plant, excluding the costs of removing inorganic/organic pollutants^5^. Meanwhile, the highly concentrated organic matter in dredged sediments is also a risk for further ecological stabilization and additional utilization^6^. Bioremediation in comparison with the costly and highly risky physical and chemical remediation processes, is a green and cost-effective technology which also can be commercially utilized in large scale^7^.

Microorganisms in sediments mediate several processes in the biogeochemical cycles of carbon, nutrients, metals, and sulfur^1^. Previous studies also suggested that Microbial Fuel Cells (MFCs) may be used for enhancing biodegradation of contaminants in anoxic environments by providing an inexhaustible source of terminal electron acceptors to a polluted environment^8^,^9^. Based on microbe–electrode interactions, various systems are being developed with time to not only remove organics, but also for the production and recovery of value-added products from substrates^10^. Another, the merging of phototrophic organisms or plants into microbial fuel cells (MFCs) is an interesting option since they can act as efficient *in situ* oxygenators, thus facilitating the cathodic reaction of microbial fuel cells^9^. Experiments have also shown that through stimulating the microbial electrogenic metabolism, dibenzothiophene removal was enhanced by more than 3-fold compared to the natural attenuation^11^. On the other hand, Zhang *et al*. arranged anodes with two different ways for enhancing the bioremediation of contaminated soil, up to 12.5% of the total petroleum hydrocarbon (TPH) was removed in reactors with anodes horizontally arranged after 135 days, which was 95.3% higher than that in the disconnected control (6.4%)^8^. Yang *et al*. built a 100 L sediment microbial fuel cells (SMFC) inoculate with heavily contaminated sediments, and the total organic chemical degradation efficiency was 22.1% in the electricity generating SMFCs, which is significantly higher than that in the open-circuited SMFC (3.8%) after two years’ long-term applicability without external electron donor addition^8^. It is worth noticing that in the past few years, considerable progresses have been made in MFC research, but significant challenges still remaining in river sediments restoration^10^,^12^.

In this study, a total volume of 195 L BMES was constructed and was expected to be employed for sediment organic removal. The aim of the present study was to supply a potential novel method to serve as an *in-situ* river sediment restoration.

## Results and Discussion

### TOC and TN variation in the sediment

**Figure 1:**
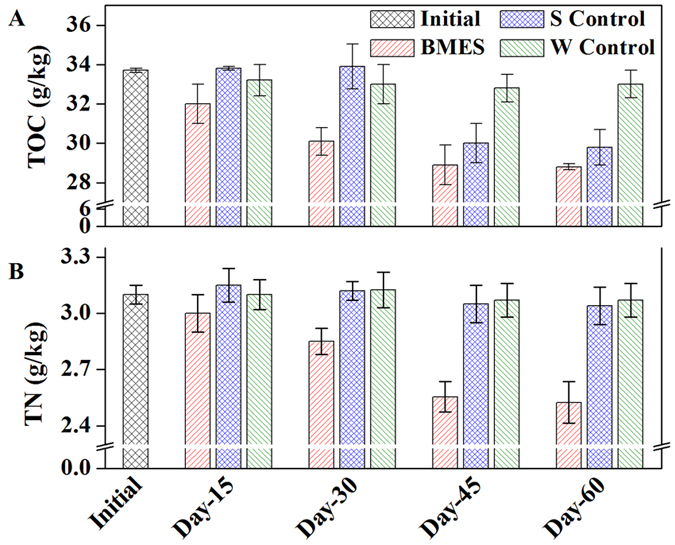
TOC and TN changes in the sediment (BMES stands for the sediment in the BMES, S Control stands for the sediment in the S Control reactor, W Control stands for the sediment in the W Control reactor).

The initial total nitrogen^13^ and the TOC of the sediment was 3.1 and 33.7 g kg^−1^ in dry sediment base (Table S1). For the BMES reactor, the TOC was decreased by 5.0% during the first 15 days of operation, and then the TOC removal was 5.6% during the second 15 days, a relative lower removal of 3.6% was obtained during the third 15 days, while it only decreased by 0.3% during the last 15 d (Total decreased by 14.5%) (Fig. 1). The BMES kept a high TOC removal during the first 45 days before the removal rate sharply declined (there was no obvious change during the last 15 days) (Table S2). During the whole operation, the TOC removal efficiency of BMES (14.5%) was 1.2- and 6.9-fold in comparison with the S Control (11.6% removal) and W Control (only 2.1% removal). It indicated that the flushing method (W Control), which was usually used for sediment restoration has little effects on the organics removal. From the experimental data obtained here, it is obviously that BMES demonstrated good capability of simultaneous organic matter removal as reported^14^,^15^.

TN decreased by 3.2% during the first 15 days of the BMES operation, and then decreased by 1.6% during the second 15 days, a removal of 12.7% was obtained during the third 15 days, while it only decreased by 1.0% during the last 15 d (Total decreased by 18.5%). But, during the same period, there were no significant changes in S Control (only 1.9% removal) and W Control (1.0% removal) during the whole operation (Table S3). The TN removal rate of the BMES was 8.7− and 18.2− fold than that of S Control and W Control.

### PAHs removal performances

**Figure 2:**
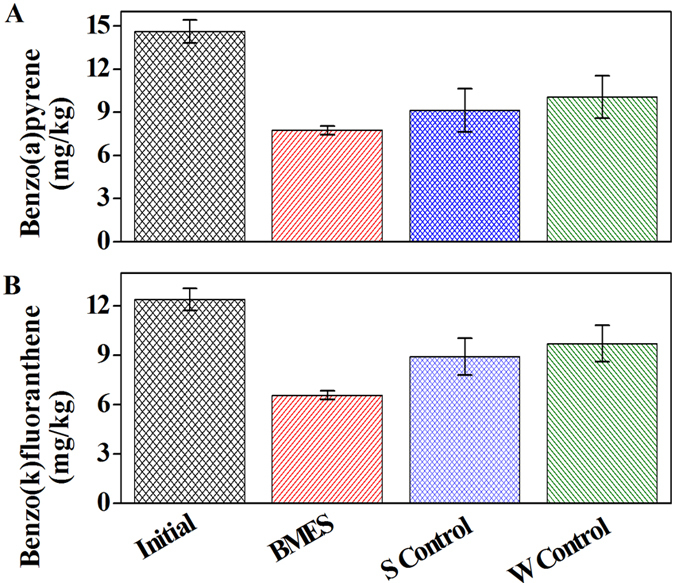
PAHs change in the sediment (BMES stands for the sediment in the BMES, S Control stands for the sediment in the S Control reactor, W Control stands for the sediment in the W Control reactor).

In total 12 kinds of PAHs were detected in the present sediments (Table 1). The concentrations of both Benzo(a) pyrene (BaP) and Benzo (k) fluoranthene (BkF) (five-ringed PAHs) was higher than 12 mg/kg, accounting for 60.35% of total PAHs (TPAHs) in the initial sediments (Fig. 2). It is probably because low molecular weight PAHs, containing two-to three-ringed PAHs were less toxic to microbes in the soil and can serve as a carbon source involved in the microbial metabolisms and accumulated more in the sediment^8^. Also high molecular weight PAHs, containing four-to six-ringed PAHs were hard to be decomposed by microbes.

The total contents of BaP during the 60 days’ operation were decreased by 50%, 37.4% and 30.8% in BMES, S Control and W Control respectively and BkF decreased by 50%, 28% and 21.8% in BMES, S Control and W Control. More important, BMES has higher PAHs removal efficiencies than that the S Control and W Control (1.4 fold PAH removal than S Control and 1.8 fold than W Control). Complex organic compounds, such as PAHs decomposition might need a long time leading to inhibition of TOC further removal in the sediment^16^ (Fig. 1). Sorption of PAHs on natural sorbents, like sands, sandy loam soils, and silt loam soils are usually normal process and is regarded as dynamic fast step compared to the biodegradation process^17^. Former research also showed that dissolved organic matter has an positive effect of PAHs sorption and induced PAHs contents are usually proportion to the dissolved organic matter^18^. PAHs in the present system was expected to be attached on the anode surface firstly and then decomposed by the anode bacteria in BMES. But in BMES, with the microbes enrichment on the anode, PAH sorption/desorption hysteresis declined, due to EPS production of anode bacteria^19^. It is obvious that the degradation was accelerated in BMES owing to the promotion by the current generated in BMES. It can also be concluded that washing flushing has no extra effects on the PAHs removal. The attached PAHs on anode was then further degraded by the electrogensis bactetria.

### TOC and TN changes in the water layer of BMES

**Figure 3:**
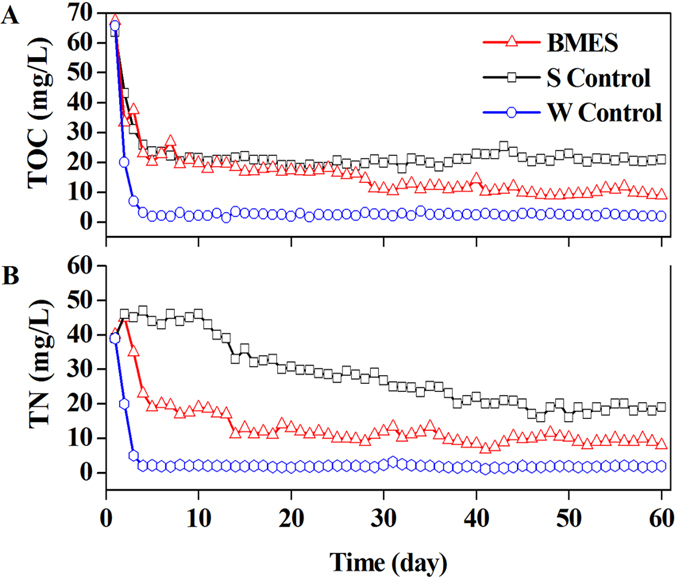
TOC and TN change in water layer.

Total 135 L tap water (TOC 1.9 mg/L) was used for the water layer, the TOC contained in the sediment began to be flushed and released into the water layer. The initial TOC concentration in water was 60 ~70 mg L^−1^, yet was reduced to <30 mg L^−1^ in the first 3 days in the three research systems (Fig. 3). Resettling of pollutants to sediments^20^,^21^ might be the main reason for the declination of TOC in the first 3 days, yet some released soluble organics from sediments remained in the water layers. In the followed 5 d to 25 d, the TOC of the BMES and S Control had similar trend. After 60 days’ operation, the TOC of the BMES was approximately half than that of the S Control. The results indicated that the deployment of electrode as electron acceptor in BMES was helpful to better removal of organic pollutants than natural biodegradation in S control. The lowest TOC contained in water layer was achieved by W Control which was washed by fresh water in a continuous flow rate of 400 L h^−1^.

After 135 L tap water (TN 1.9 mg/L) was used for the water layer, the initial concentration of TN in the water was 39 to 40 mg L^−1^ for the flushing and also the desorption. During the first 3 days, TN content in BMES and W Control dropped sharply, and in the later 30 days experiments, TN removal rates in BMES and W Control slowed down and kept at 7.47~13.3 mg L^−1^ and 1.1 ~3.2 mg L^−1^ respectively. In the first 3 days, TN in S control was at 39–45 mg L^−1^ and then at 17–28 mg L^−1^ in the following experimental period.

### DO, pH and EC changes in the water layer of BMES

**Figure 4:**
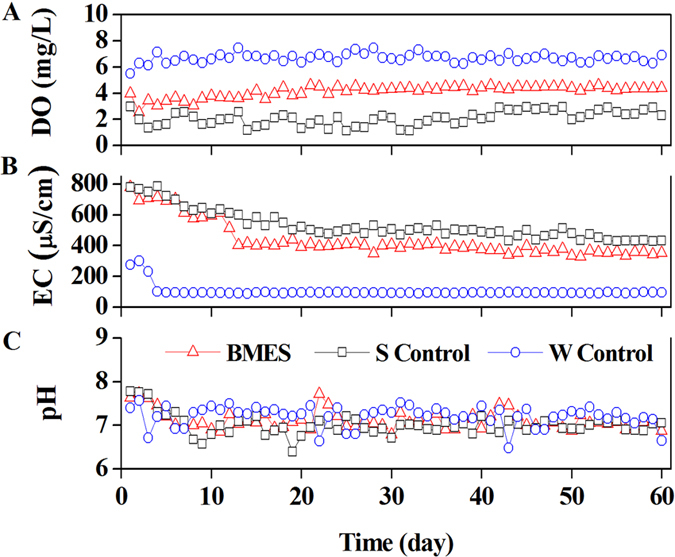
DO, pH, and EC changes in water layer.

As the reaction proceeded, the DO in the BMES, S Control and W Control maintained a relatively stable range (4.3 ± 0.082 mg L^−1^, 2.6 ± 0.21 mg L^−1^ and 6.5 ± 0.1 mg L^−1^). Higher DO in W Control should be related to the flushing water and keep the DO concentration similar to the DO in normal water (Fig. 4). The DO in BMES was 2 times higher than that in S control. In this study, changes in pH were observed for the entire duration. The experimental results clearly indicate that the pH of three reactors showed similar performance, and all were stable at 6.8–7.4. For the BMES, water conductivity was relatively constant at 135 μs cm^−1^, which was similar to that in the SMFC systems using fresh water as the electrolyte. This indicates a relatively low conductivity compared to the BMFC reported previously^22^,^23^,^24^.

The decomposition of organic would usually have robbed the dissolved oxygen from water which might deteriorate water quality. It is significant that the higher DO in BMES meant less DO was consumed in TOC or TN removal in BMES. When the EC of the three reactors were steady, W Control was 4- and 5-fold lower than the BMES and Control, respectively. It is significant that EC had the same trend with the TOC in the surface water. The difference on conductivity reduced with the decrease of TOC^25^.

### Power generation in BMES

**Figure 5:**
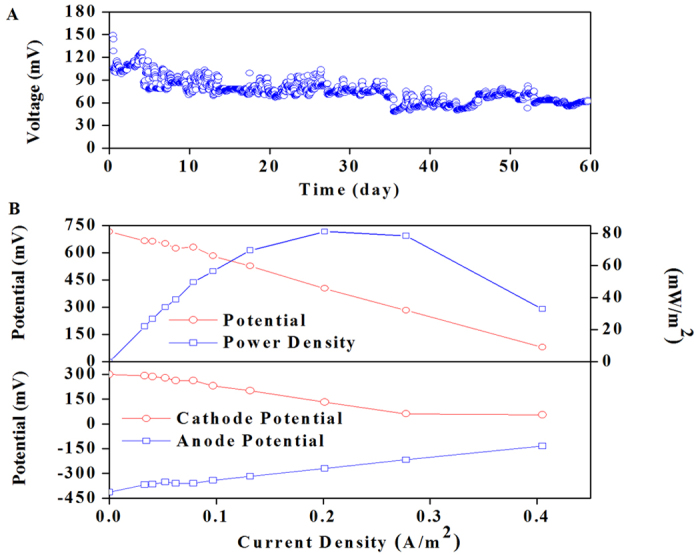
Performance of the BMES: (**A**) Representative time course of cell voltage for the BMES (**B**) Polarization and power curves showing the fuel-cell performances of BMES anode and cathode polarizationcurves.

Under the closed circuit (CC) mode, an average voltage of 90 mV was initially achieved in the BMES, although some voltage fluctuations were observed (Fig. 5A). The voltage decreased to 65 mV on day 11. In the present study, the internal resistance evaluated by the polarization slope method was about 40 Ω. This showed that the scale of mass transport limitations influenced the BMES performance, predominantly at the anode electrode. The potential and power density, as a function of current density, were obtained as shown in Fig. 5B. Based on the power curve, the maximum power density for the BMES was 81 mW m^−2^ at day 20, which is relatively higher compared with that in previous studies^26^.

For the BMES, the anode potential varied over a narrow range of −410 to −440 mV, while the cathode potential covered a much wider range of 240 to 180 mV, possibly indicating that its current was limited by the cathode or external resistance (Fig. 5C).

A power management system (PMS) was designed that enables BMES to drive 9 red LEDs in parallel^27^. Six independent capacitor-based circuits were used to harvest electrical energy from the BMES. Each circuit consisted of capacitors (3.3 F, Panasonic Corporation, Japan) and relays controlled by programmable microcontroller (XD-J16H, Xunda Corporation, China). The capacitors in each circuit were charged in parallel by the corresponding module (1 min) and then charged capacitors connected in series to discharge (1 min) (Movie S1). Our results demonstrate that BMES can be a viable alternative renewable power source (Fig. S2).

There are also strong correlations between the current output of a simple anode-resistor-cathode device and rates of anaerobic microbial activity (TOC content) in a diversity of anoxic sediments^28^. At the initial stage of deployment, the BMES could obtain high voltage outputs, but over a long operational period, nutrient depletion, mass transfer resistance, and anode/cathode fouling, could lower the system performance^29^,^30^. Several factors, such as spatial and temporal variability, natural fluctuations, temperature changes, pressure variations, water flow changes, salinity and conductivity changes, and dissolved oxygen affect the BMES functions^29^. The kinetics of electron transfer from microorganisms to the anode were mainly restricted by the anode potential. Overall, the BMES revealed a high cathode potential, the lowest anode potential, and the highest cell potential (189–591 mV)^7^.

### Community analysis

Analysis of the anodic bacterial community using 16S rDNA gene pyrosequencing revealed the enrichment of genera with potential exoelectrogenic capability^31^. And this has significant implications in determining the microbial community structure in SMFCs in each aquatic environment and consequently the fate and removal kinetics of organic pollutants in contaminated sediments^32^. The Shannon diversity index provided the species richness (i.e., the number of species present) and distribution of each species (i.e., the evenness of the species) among all the species in the community^33^. BMES had the highest diversity (Shannon = 7.07) that was slightly larger than that of Initial (Shannon = 6.87) and S Control (Shannon = 6.50) among the 4 communities, while S Control had the lowest diversity (Shannon = 6.09).

**Figure 6:**
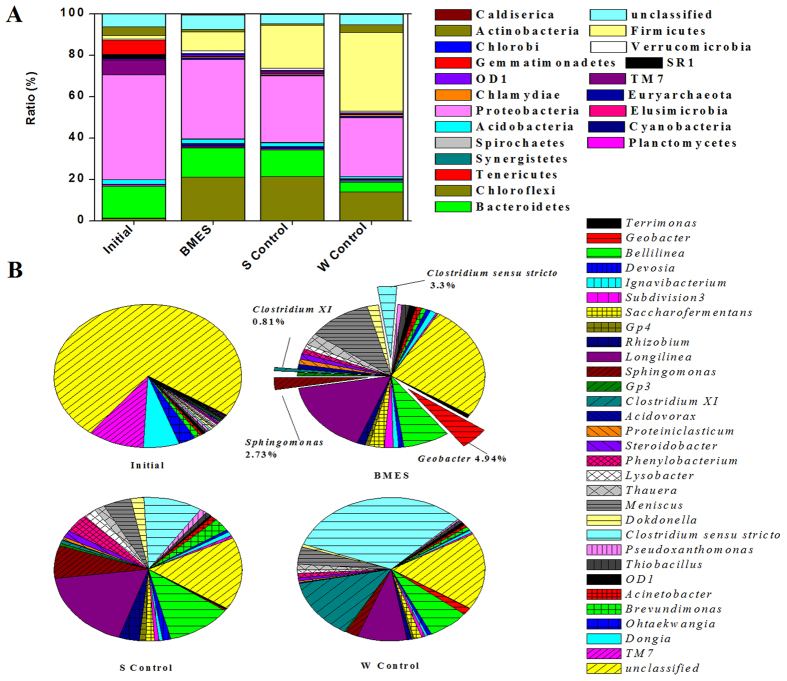
Analyses of sequencing data for bacterial communities. (**A**) Relative abundances of major phylum-level bacterial groups estimated using the RDP classifier. (**B**) Relative abundances of major genus-level bacterial groups estimated using the RDP classifier.

Based on the sequencing results (Fig. 6), the communities analyzed Initially composed of Gammaproteobacteria (50.38%), Bacteroidetes (15.33%), and TM7 (7.37%). Bacteroidia (18.8%), Proteobacteria (32.05%), Chloroflexi (21.4%), and Firmicutes (20.75%) were detected in the S Control. W Control was colonized by Firmicutes (37.93%), Proteobacteria (28.27%), and Chloroflexi (13.9%). For the BMES, Bacteroidia (14.1%), Proteobacteria (38.4%), Chloroflexi (21.04%), and Firmicutes (9.22%) were detected. Deltaproteobacteria was also believed to be responsible for the electron transfer to the electrode and were also detected in Initial, BMES, S Control, and W Control with 1.95%, 12.26%, 4.89% and 11.89% dominance, respectively, which differs slightly from the values reported in two previous studies at 10% and 70%, respectively^12^,^34^. *Geobacter* was also detected abundantly in these soil samples, representing approximately 0.07%, 4.94%, 0.16% and 0.12% of the total bacteria in Initial, BMES, S Control, and W Control respectively. The ratios of Geobacteraceae sequences were previously reported to have increased from 0.13 to 0.74% in response to an increase in current density in rhizosphere MFC^35^. Here, *Geobacter* sequences were more abundant in anode-associated soil than in bulk soil, suggesting that they were involved in electricity generation in the BMES.

Earlier studies have observed that *Gammaproteobacteria* and *Bacteroidetes* occurred at high numbers within libraries from electrode biofilms, in which the *Gammaproteobacteria* were believed to be responsible for electron transfer and consequently power generation^23^,^34^. *Flavobacteria* and *Bacteroidia* have earlier been identified as the dominant bacteria for electricity generation^23^,^36^ (Fig. 6). The microbial community composition observed in this study shows that the microbial community in BMES was dominated by exoelectrogenic bacteria^37^,^38^. A number of less prevalent bacteria were also detected (Fig. 6)^39^. The variety of the bacterial colonies in sediments is attributed to the process of enrichment of electricity bacteria in anodes^36^.

## Methods

### BMES construction and operation

A 195 L of the Benthic Microbial Electrochemical Systems (BMES) was constructed using a three-dimensional anode (Tri-TDA) and floating air cathode (FAC). The BMES consisted of a 1.20 × 0.25 m plastic channel with a depth of 0.65 m. Sediment from Ash River, Heilongjiang Province was taken and used in this research. A sediment layer of 15 cm was placed in the bottom of the BMES (Total volume of sediment was 45.0 L). Water depth was 45 cm, and the total volume of water layer was 135 L. The floating air-cathode (0.1 m^2^) was designed using rolling pressure activated carbon as a catalyst layer and stainless steel mesh (SSM) in the middle for electron collection and transfer^40^. The three-dimensional anode (Fig. S1B) was constructed using carbon mesh heat-pressure adhered to ‘honeycomb’ structure supports. The TDA (with height of 10 cm) was stood in the sediment of BMES, and full covered with river sediments. The distance between the TDA and FAC was connected by titanium wire with a 10 Ω external resistor. No other inoculum was used in the system and the entire experiment only relied on naturally occurring bacteria in the sediments.

Two controls were set in this research with the same size and structure of the BMES as above mentioned. S Control was designed without electrodes fabrication and operated under the same conditions as BMES to compare pollutant removal efficiencies. W Control was designed not only with no electrodes fabrication but also washed by fresh water in a continuous flow rate of 400 L h^−1^ to investigate the organic removal efficiencies in contrast to the BMES.

### Experiments and sampling

Experiments were performed in a batch mode over a period of 60 d. Water samples were taken daily for TOC, TN, pH, EC (Electrical conductivity) and DO (Dissolved oxygen) monitoring. Sediment samples were taken every 15 days for TOC, TN detection, and PAHs in sediments were quantified after 60 days’ operation.

Sediment samples were collected from the BMES, S Control and W Control using gravity core sampler. Two sampling spots were set in the middle along the width direction and 40 cm and 80 cm from the influent point along the long direction (Fig. S1C). Water Sampling points for water body analysis were placed at the side of the reactor, and two points were set in the vertical direction away 10 cm and 30 cm below the water surface. Samples were taken out from the reactors for further analysis (Fig. S1A).

### Analysis and calculation methods

The voltages were recorded every 30 minutes using a data acquisition board (PISO-813.ICP DAS CO, Ltd) connected to a personal computer and converted to power according to Ohm’s law, P = *IU*, where, P = power (W), I = current (A), and *U* = voltage (V). Anode and cathode potentials were measured against an Ag/AgCl reference electrode (Model 13-620-45; Fisher Scientific; Hampton, NH) suspended in the overlying water. For electrochemical processes such as MES, the most relevant production rate is thatnormalized byelectrode surface area, for example gm^−2^ d^−1^, due to the limitation of electrode surface for directing these processes^41^. Power density (mW/m^2^) and current density (mA/m^2^) were calculated with the function of cathodic surface area (m^2^). In order to estimate the energy produce by MFC during the experiment, ∆ V acquired by data logger was used to calculate the current (I) from Ohms law: I = V·R^−1^. Tus, energy produced by BMES during 60 days operation was calculated as kilowatt hour per cubic meter of influent wastewater (kWh m^–3^) by the following equation:


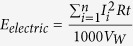


Analysis of TOC, TN and nitrate nitrogen was carried out according to APHA standard methods (APHA, 1998). Electrical conductivity (EC), pH and Dissolved oxygen (DO) were monitored using hand-held meters during the research period. Inductively coupled plasma-mass spectrometry (ICP-MS, Agilent 7500, Agilent, USA) was applied for determination of the heavy metals in this work. In order to know the PAH (Polycyclic aromatic hydrocarbons) contents and removal efficiencies in the sediments, PAHs were analyzed using external standards method. A standards contained twelve 2− to 6− ring PAHs were used in this study (Table S4). Accelerated Solvent Extractor system (ASE 350 System, Thermo Scientific™ Dionex™, Waltham, MA) was used to extract the PAHs from sediment samples.

### Microbial Community Analysis

The total community DNA in the sediment was directly extracted from the 0.25 g homogenized samples using the Omega D5625-01 Soil DNA Kit (Omega Bio-Tek, America) according to the manufacturer’s instructions. Bacterial 16S rDNA was amplified from total DNA using 63 F forward (50-CAGGCCTAACACATGCAAGTC-30) and 1387 R reverse (30-GGGCGGWGTGTACAAGGC-50) primers. In the present metagenomic analysis, a single lane of the Illumina Hiseq 2000 sequencer was used for the biofilm and soil samples. Genes with BLAST hits to the NCBI-NR database (BLAST-hit genes) were subjected to MEGAN analysis to predict their taxonomic distribution. Initial soil samples from Ash River was also used as control to compare the community changes in the system.

## Additional Information

**How to cite this article**: Li, H. *et al*. A Pilot-scale Benthic Microbial Electrochemical System (BMES) for Enhanced Organic Removal in Sediment Restoration. *Sci. Rep.* **7**, 39802; doi: 10.1038/srep39802 (2017).

**Publisher's note:** Springer Nature remains neutral with regard to jurisdictional claims in published maps and institutional affiliations.

## Supplementary Material

Supplementary Information

Supplementary Movie S1

## Figures and Tables

**Table 1:** The content and ratio of different PAHs in the initial sediments from Ash River.

PAH	Content (mg/kg)	Ratio (%)	Molecular formula
Phenanthrenes (Phe)	2.851882	6.3691	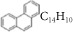
Anthracene (Ant)	2.750238	6.1421	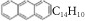
Fluoranthene (Flu)	0.005998	0.0134	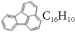
Pyrene (Pyr)	0.278455	0.6219	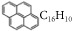
Benzo(a) anthracene (BaA)	1.245569	2.7817	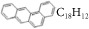
Chrysene^42^	2.218675	4.955	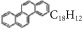
Benzo (b) fluoranthene (BbF)	3.330342	7.4377	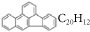
DiBenzo(a, h) anthracene (DaA)	0.205908	0.4599	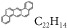
Benzo (g, h, i) perylene (BghiP)	2.367114	5.2865	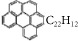
Indeno (1,2,3-c, d) pyrene (Ind)	2.493461	5.5687	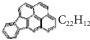
Benzo (k) fluoranthene (BkF)	12.42516	27.7493	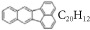
Benzo(a) pyrene (BaP)	14.60375	32.6147	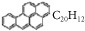

## References

[b1] Martins G., Peixoto L., Brito A. G. & Nogueira R. Phosphorus–iron interaction in sediments: can an electrode minimize phosphorus release from sediments? Reviews in Environmental Science and Bio/Technology. 13, 265–275 (2014).

[b2] Martins G. . Impact of an external electron acceptor on phosphorus mobility between water and sediments. Bioresour Technol. 151, 419–423 (2014). 24210650 10.1016/j.biortech.2013.10.048

[b3] Beg M. U. . Chemical contamination and toxicity of sediment from a coastal area receiving industrial effluents in Kuwait. Arch Environ Contam Toxicol. 41, 289–297 (2001). 11503065 10.1007/s002440010251

[b4] Bolam S. G. . Ecological consequences of dredged material disposal in the marine environment: a holistic assessment of activities around the England and Wales coastline. Mar Pollut Bull. 52, 415–426 (2006). 16256147 10.1016/j.marpolbul.2005.09.028

[b5] Liu F. H. . Bacterial and archaeal assemblages in sediments of a large shallow freshwater lake, Lake Taihu, as revealed by denaturing gradient gel electrophoresis. J Appl Microbiol. 106, 1022–1032 (2009). 19191955 10.1111/j.1365-2672.2008.04069.x

[b6] Song T.-s., Tan W.-m., Wu X.-y. & Zhou C. C. Effect of graphite felt and activated carbon fiber felt on performance of freshwater sediment microbial fuel cell. Journal of Chemical Technology & Biotechnology. 87, 1436–1440 (2012).

[b7] Zhao J. . Electricity generation from Taihu Lake cyanobacteria by sediment microbial fuel cells. Journal of Chemical Technology & Biotechnology. 87, 1567–1573 (2012).

[b8] Zhang Y. . Horizontal arrangement of anodes of microbial fuel cells enhances remediation of petroleum hydrocarbon-contaminated soil. Environ Sci Pollut Res Int. 22, 2335–2341 (2015). 25189807 10.1007/s11356-014-3539-7

[b9] ElMekawy A., Hegab H. M., Vanbroekhoven K. & Pant D. Techno-productive potential of photosynthetic microbial fuel cells through different configurations. Renewable and Sustainable Energy Reviews. 39, 617–627 (2014).

[b10] Pandey P. . Recent advances in the use of different substrates in microbial fuel cells toward wastewater treatment and simultaneous energy recovery. Applied Energy. 168, 706–723 (2016).

[b11] Rodrigo J., Boltes K. & Esteve-Nunez A. Microbial-electrochemical bioremediation and detoxification of dibenzothiophene-polluted soil. Chemosphere. 101, 61–65 (2014). 24332730 10.1016/j.chemosphere.2013.11.060

[b12] Li W. W. & Yu H. Q. Stimulating sediment bioremediation with benthic microbial fuel cells. Biotechnol Adv. 33, 1–12 (2015). 25560929 10.1016/j.biotechadv.2014.12.011

[b13] Buntner D., Spanjers H. & van Lier J. B. The influence of hydrolysis induced biopolymers from recycled aerobic sludge on specific methanogenic activity and sludge filterability in an anaerobic membrane bioreactor. Water Res. 51, 284–292 (2014). 24284260 10.1016/j.watres.2013.10.065

[b14] Yang Y. . Enhancing the bioremediation by harvesting electricity from the heavily contaminated sediments. Bioresour Technol. 179, 615–618 (2015). 25549820 10.1016/j.biortech.2014.12.034

[b15] Zhou Y. L. . To improve the performance of sediment microbial fuel cell through amending colloidal iron oxyhydroxide into freshwater sediments. Bioresour Technol. 159, 232–239 (2014). 24657753 10.1016/j.biortech.2014.02.082

[b16] Arends J. B. . Greenhouse gas emissions from rice microcosms amended with a plant microbial fuel cell. Appl Microbiol Biotechnol. 98, 3205–3217 (2014). 24201892 10.1007/s00253-013-5328-5

[b17] Barret M., Carrère H., Patau M. & Patureau D. Kinetics and reversibility of micropollutant sorption in sludge. Journal of Environmental Monitoring. 13, 2770 (2011). 21860870 10.1039/c1em10181a

[b18] Yang X.-H. . PAHs Sorption and Desorption on Soil Influenced by Pine Needle Litter-Derived Dissolved Organic Matter. Pedosphere. 24, 575–584 (2014).

[b19] Zhang J. & He M. Effect of dissolved organic matter on sorption and desorption of phenanthrene onto black carbon. Journal of Environmental Sciences. 25, 2378–2383 (2013). 10.1016/s1001-0742(12)60328-3 24649667

[b20] Owabor C. N. . Comparative Study of the Adsorption and Desorption Behavior of Single and Multi-Ring Aromatics in Sediment Fractions. Advances in Chemical Engineering and Science. 03, 67–73 (2013).

[b21] Colombo S. d. M. & Masini J. C. Developing a fluorimetric sequential injection methodology to study adsorption/desorption of glyphosate on soil and sediment samples. Microchemical Journal. 98, 260–266 (2011).

[b22] Deng H. . Factors Affecting the Performance of Single-Chamber Soil Microbial Fuel Cells for Power Generation. Pedosphere. 24, 330–338 (2014).

[b23] Zhang Y., Min B., Huang L. & Angelidaki I. Generation of electricity and analysis of microbial communities in wheat straw biomass-powered microbial fuel cells. Appl Environ Microbiol. 75, 3389–3395 (2009). 19376925 10.1128/AEM.02240-08 PMC2687294

[b24] An J., Lee S. J., Ng H. Y. & Chang I. S. Determination of effects of turbulence flow in a cathode environment on electricity generation using a tidal mud-based cylindrical-type sediment microbial fuel cell. J Environ Manage. 91, 2478–2482 (2010). 20688427 10.1016/j.jenvman.2010.06.022

[b25] Li X. . Salinity and Conductivity Amendment of Soil Enhanced the Bioelectrochemical Degradation of Petroleum Hydrocarbons. Sci Rep. 6, 32861 (2016). 27597387 10.1038/srep32861 PMC5011858

[b26] Wang D.-B. . Electricity generation from sediment microbial fuel cells with algae-assisted cathodes. International Journal of Hydrogen Energy. 39, 13224–13230 (2014).

[b27] Dong Y. . A 90-liter stackable baffled microbial fuel cell for brewery wastewater treatment based on energy self-sufficient mode. Bioresour Technol. 195, 66–72 (2015). 26096580 10.1016/j.biortech.2015.06.026

[b28] Wardman C., Nevin K. P. & Lovley D. R. Real-time monitoring of subsurface microbial metabolism with graphite electrodes. Front Microbiol. 5, 621 (2014). 25484879 10.3389/fmicb.2014.00621 PMC4240160

[b29] Karra U. . Stability characterization and modeling of robust distributed benthic microbial fuel cell (DBMFC) system. Bioresour Technol. 144, 477–484 (2013). 23890975 10.1016/j.biortech.2013.06.104

[b30] Wang H. . Cascade degradation of organic matters in brewery wastewater using a continuous stirred microbial electrochemical reactor and analysis of microbial communities. Sci Rep. 6, 27023 (2016). 27270788 10.1038/srep27023 PMC4895234

[b31] Hamdan H. Z. . Assessment of the performance of SMFCs in the bioremediation of PAHs in contaminated marine sediments under different redox conditions and analysis of the associated microbial communities. Sci Total Environ (2016). 10.1016/j.scitotenv.2016.09.232 27720249

[b32] Luo H. . Effect of *in-situ* immobilized anode on performance of the microbial fuel cell with high concentration of sodium acetate. Fuel. 182, 732–739 (2016).

[b33] Zheng B., Wang L. & Liu L. Bacterial community structure and its regulating factors in the intertidal sediment along the Liaodong Bay of Bohai Sea, China. Microbiological Research. 169, 585–592 (2014). 24231160 10.1016/j.micres.2013.09.019

[b34] Ewing T. . Scale-up of sediment microbial fuel cells. Journal of Power Sources. 272, 311–319 (2014).

[b35] Kouzuma A., Kaku N. & Watanabe K. Microbial electricity generation in rice paddy fields: recent advances and perspectives in rhizosphere microbial fuel cells. Appl Microbiol Biotechnol. 98, 9521–9526 (2014). 25394406 10.1007/s00253-014-6138-0

[b36] Lin H., Wu X., Miller C. & Zhu J. Improved performance of microbial fuel cells enriched with natural microbial inocula and treated by electrical current. Biomass and Bioenergy. 54, 170–180 (2013).

[b37] Kouzuma A. . Comparative metagenomics of anode-associated microbiomes developed in rice paddy-field microbial fuel cells. PLoS One. 8, e77443 (2013). 24223712 10.1371/journal.pone.0077443 PMC3815305

[b38] Alatraktchi F. A. a., Zhang Y. & Angelidaki I. Nanomodification of the electrodes in microbial fuel cell: Impact of nanoparticle density on electricity production and microbial community. Applied Energy. 116, 216–222 (2014).

[b39] Pisciotta J. M. . Enrichment of microbial electrolysis cell biocathodes from sediment microbial fuel cell bioanodes. Appl Environ Microbiol. 78, 5212–5219 (2012). 22610438 10.1128/AEM.00480-12 PMC3416445

[b40] Dong H. . A novel structure of scalable air-cathode without Nafion and Pt by rolling activated carbon and PTFE as catalyst layer in microbial fuel cells. Water Res. 46, 5777–5787 (2012). 22939222 10.1016/j.watres.2012.08.005

[b41] Patil S. A. . A logical data representation framework for electricity-driven bioproduction processes. Biotechnol Adv. 33, 736–744 (2015). 25765230 10.1016/j.biotechadv.2015.03.002

[b42] Stams A. J. . Exocellular electron transfer in anaerobic microbial communities. Environ Microbiol. 8, 371–382 (2006). 16478444 10.1111/j.1462-2920.2006.00989.x

