# Supplementary material for: A Pilot-scale Benthic Microbial Electrochemical System (BMES) for Enhanced Organic Removal in Sediment Restoration
Source: Sci Rep. 2017 Jan 6;7:39802. doi: 10.1038/srep39802 (PMC5216391; doi:10.1038/srep39802)
Supplement: Supplementary Information [file srep39802-s1.doc]

**Appendix A. Supplementary data**

**A** **Pilot-scale Benthic Microbial Electrochemical System (BMES) for Enhanced Organic Removal in Sediment Restoration**

Henan Li^a^, Yan Tian ^b **^, Youpeng Qu^a,c^, Ye Qiu^a^, Jia Liu^a^ & Yujie Feng ^a,*^

^a^State Key Laboratory of Urban Water Resource and Environment, Harbin Institute of Technology. No 73 Huanghe Road, Nangang District, Harbin 150090, China;

^b^ Heilongjiang Academy of Chemical Engineering, No 3, Nanhu Street, Century District, High-Tech Zone, Harbin 150028, Heilongjiang, China

^c^School of Life Science and Technology, Harbin Institute of Technology. No. 2 Yikuang Street, Nangang District, Harbin 150080, China

*****Corresponding Author:

E-mail: [yujief@hit.edu.cn](mailto:yujief@hit.edu.cn); phone: (+86)451-86287017;

Fax: (+86) 451-86287017

******Co-corresponding Author:

E-mail: hhytianyan@163.com

Number of figures: 2

Number of tables: 4

Table S1 The Characteristics of the Initial Sediment

|  | Value |
| --- | --- |
| TOC | 33.7 g kg^−1^ |
| TN | 3.1 g kg^−1^ |
| pH | 7.2 |
| EC | 500 μs/cm |

Table S 2 The TOC content change in sediment

| TOC (%) | Value  (g kg^−1^) | Removal rate (%) |
| --- | --- | --- |
| Initial | 3.37±0.01 |  |
| BMES-15 | 3.20±0.10 | 5.04 |
| S Control-15 | 3.38±0.01 | -0.30 |
| W Control-15 | 3.32±0.08 | 1.48 |
| BMES-30 | 3.01±0.17 | 10.68 |
| S Control-30 | 3.39±0.19 | -0.59 |
| W Control-30 | 3.30±0.23 | 2.08 |
| BMES-45 | 2.89±0.10 | 14.24 |
| S Control-45 | 3.00±0.10 | 10.98 |
| W Control-45 | 3.28±0.07 | 2.67 |
| BMES-60 | 2.88±0.04 | 14.54 |
| S Control-60 | 2.98±0.14 | 11.57 |
| W Control-60 | 3.30±0.44 | 2.08 |

Table S 3 The TN content change in sediment

| TN (%) | Value  (g kg^−1^) | Removal Rate (%) |
| --- | --- | --- |
| Initial | 0.31±0.005 |  |
| BMES-15 | 0.30±0.010 | 3.23 |
| S Control-15 | 0.315±0.009 | -1.61 |
| W Control-15 | 0.31±0.008 | 0.00 |
| BMES-30 | 0.29±0.012 | 4.84 |
| S Control-30 | 0.31±0.012 | -0.81 |
| W Control-30 | 0.313±0.015 | 3.23 |
| BMES-45 | 0.26±0.008 | 17.58 |
| S Control-45 | 0.31±0.010 | 1.61 |
| W Control-45 | 0.31±0.009 | 0.97 |
| BMES-60 | 0.25±0.011 | 18.50 |
| S Control-60 | 0.30±0.003 | 1.94 |
| W Control-60 | 0.31±0.003 | 2.26 |

Table S 4 The heavy metal content change in sediment

|  | Cd | As | Co | Cr | Cu | Ni | Pb |
| --- | --- | --- | --- | --- | --- | --- | --- |
| Value  (mg/kg) | 0.525±0.025 | 0 | 8.53±0.28 | 78.39±1.24 | 41.95±2.15 | 21.13±1.23 | 12.2±5.15 |

We had detected the concentration of some heavy metals in the river sediment before the operation with ICP-AES, while we didn’t find any high contained heavy metals in the sediment.


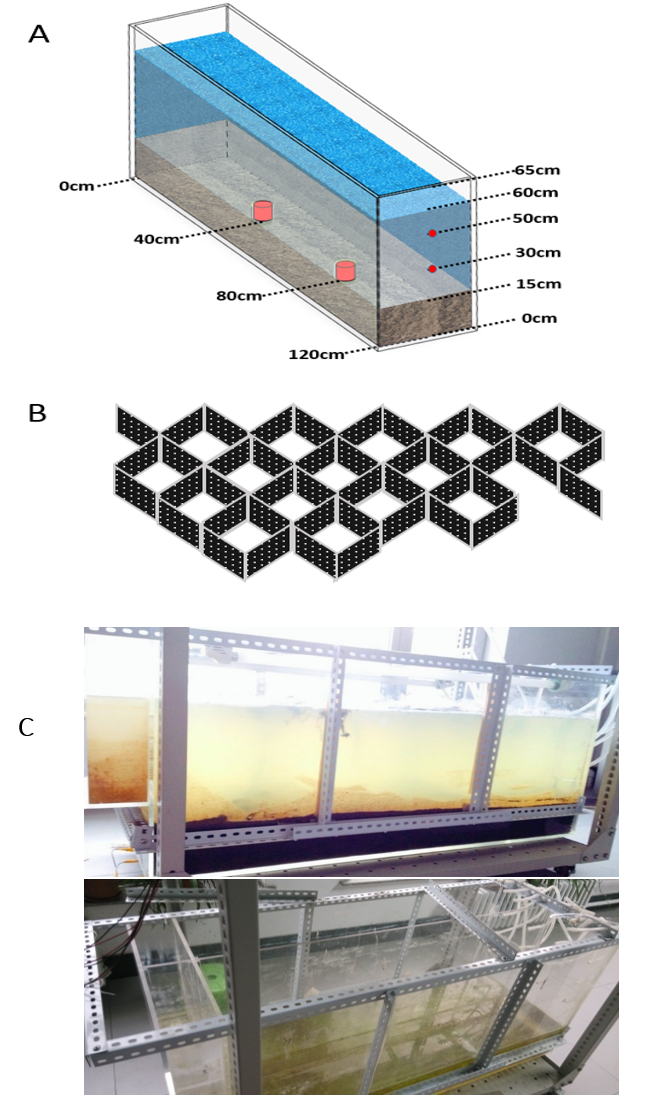


Fig. S1 Sampling points and anode structure. (A) Sampling points for sediment and water body. The yellow parts shows the sampling points for sediment, the red parts shows the sampling points for the water body. (B) Structure diagram of the anode. (C) the picture of cell.


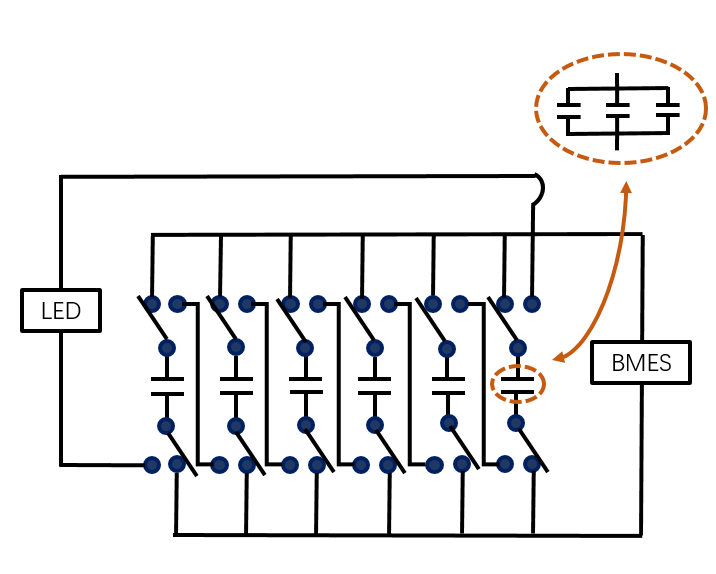


Fig.S2 A schematic diagram of the power management system (PMS).

**Energy recovery of the BMES**

Energy generation (E) was expressed as kilowatt hour per cubic meter of influent wastewater (kWh m^–3^) according to formula (1).

$E_{electric}=\frac{\sum_{i=1}^{n} I_{i}^{2}Rt}{1000V_{W}}$ (1)

I_1_: mean current of cell; R: external resistor of each cell, 10 Ω; t: 0.5 h; *V*_w_: total working volume of the BMES, 350× 10^–3^ m^3^.

Then the total power generation was 0.25 kWh m^–3^ during 60 days operation.

**Video caption**

A power management system (PMS) was designed that enables BMES to drive 9 red LEDs in parallel [^1^](#_ENREF_1). Six independent capacitor-based circuits were used to harvest electrical energy from the BMES. Each circuit consisted of capacitors (3.3 F, Panasonic Corporation, Japan) and relays controlled by programmable microcontroller (XD-J16H, Xunda Corporation, China). The capacitors in each circuit were charged in parallel by the corresponding module (1 min) and then charged capacitors connected in series to discharge (1 min) (Movie.S1).

1. Dong, *Y. et a*l. A 90-liter stackable baffled microbial fuel cell for brewery wastewater treatment based on energy self-sufficient mode*. Bioresour Techno*l**. 1**95, 66-72 (2015).
